# What do specialist mental health professionals think of the mental health services for people with intellectual disabilities in Singapore?

**DOI:** 10.1177/17446295211030094

**Published:** 2021-08-24

**Authors:** Jonathan Ee, Biza Stenfert Kroese, Jan Mei Lim, John Rose

**Affiliations:** University of Birmingham, UK; University of Birmingham, UK; Institute of Mental Health, Singapore; University of Birmingham, UK

**Keywords:** intellectual disabilities, qualitative research, mental health professionals, staff attitudes, mental health services

## Abstract

**Background:**

This research aimed to investigate the views and experiences of specialist mental health professionals working with adults with intellectual disabilities and mental health problems in Singapore in order to gain insight into the functioning of the local specialist intellectual disability mental health service and how it may be improved.

**Methods:**

Eight staff members from specialist service were interviewed. The transcriptions of the interviews were analysed using thematic analysis.

**Results:**

Analysis revealed four themes (1) Identifying their roles; (2) Ensuring continuity of care; (3) Disempowerment of service users and (4) Improving clinical practice.

**Conclusions:**

Participants identified the challenges they faced working with this population. They highlighted the importance of building therapeutic relationships during the treatment process and discussed the stigma that people with intellectual disabilities face in the community. Recommendations and implications are discussed in relation to service provision, improving staff knowledge and recruiting more staff to work in this field.

## Introduction

There are differing views regarding the most effective service models for people with intellectual disabilities and mental health problems (Davis et al., 2008; Sheehan et al., 2013). Mainstream health services have increased their accessibility for this group in line with most western countries’ governmental policies that encourage community integration, including the provision of community as opposed to specialist intellectual disability health services (Al-Zboon and Hatmal, 2016; Gill et al., 2002). However, debate continues regarding the provision of mental health services. Chaplin (2004) noted that people with intellectual disabilities receive a poorer standard of care and experience worse outcomes compared to the general population when accessing specialist intellectual disability mental health services. Adults with intellectual disabilities often experience long periods of hospitalisation and restrictive practices, including the overuse of psychotropic medication, are common treatment approaches to deal with their mental health problems (Bowring et al., 2017; Chaplin, 2011). Some countries including the UK and Canada have established that specialist services improve the standard of treatment for people with intellectual disabilities (Turnbull, 1995) and result in more positive outcomes for them, such as reduction in inpatient admissions (Richings et al., 2011) and less institutionalisation (Xenitidis et al., 2004).

There is emerging research that explores the views and experiences of mental health professionals (MHPs) working in specialist intellectual disability mental health services (Ee et al., 2021a, 2021b). The views of intellectual/learning disability nurses have been explored by Doody et al. (2018) who made suggestions for improving mainstream services through training and education. Other research on service providers’ perspectives reported a shortage of specialist intellectual disability mental health services, a lack of coordination between different services and clients being excluded from mainstream services (Scior and Grierson, 2004).

Much of the research literature is focused on western perspectives and very little is known about MHPs working in specialist intellectual disability mental health services in Asian countries. Most of the studies conducted within the Asian context are quantitative and focus solely on medical staff, ignoring the views of other MHPs from other professions (Lin et al., 2009; Sajith et al., 2017; Wong and Wong, 2008). The views of the support staff and carers have been explored in Hong Kong but not as yet the views of MHPs (Wang et al., 2011; Wong and Wong, 2008). There is a lack of qualitative studies conducted in Singapore and other Asian countries to provide an insight into the experiences of MHPs working in specialist mental health services (Kwok, 2001).

### Background of Singapore culture

There are many unique characteristics about Singapore compared to other South East Asia or Asian countries. The majority of the population use English as their first spoken language. Many of the political, social and healthcare institutions have evolved from past colonial British systems with a Western perspective. As such, Singapore shares similarities with the British healthcare system, using modern Western medicine as a treatment approach to address the general healthcare and mental health needs of the population. Singaporeans have easy access to healthcare and education facilities that results in better subjective well-being, higher quality of life and a longer life expectancy when compared to other countries in the region (Chan and Kamala Devi, 2012; Leow et al., 2013). With the advancement in economic progress following independence, Singaporeans are becoming more affluent, technologically advanced and exposed to Western values of individualism. Strong economic success has led Singapore to become a developed country that shares many similarities with Western developed countries and societies.

Nevertheless, Singapore has its own distinct culture as it is also influenced by non-western philosophies due to its proximity to China and other South East Asia countries. Singaporeans endorse social cohesiveness, belonginess to families and communities that look after one another (Kee, 2004). Many commonly held values are based on Chinese Confucian principles that emphasise the importance of hierarchical structures and relationships as a way of order in a society (Foo et al., 2006). Hence, Singaporeans have learned to defer respect to people in authority, display conformity towards societal norms and place less emphasis on individualistic needs and values. This provides a juxtaposition of ‘East meets West’ identity among Singaporeans who have adopted features of collectivistic values in a metropolitan state.

### Singapore healthcare services

The healthcare system in Singapore purports to ensure that general health services remain affordable and accessible for its citizens. Singaporeans pay subsidised rates when they receive medical treatment at polyclinics and the physicians will refer them for tertiary general health or mental health services when their medical conditions become too complex and difficult to manage in community services.

There is an emerging shift initiated by the Singapore Ministry of Health towards expanding mental health community-based services, provided in government polyclinics or private general practitioner clinics, hence increasing the accessibility to care and reducing the demand for institutionalised care (Kua and Rathi, 2019). High levels of resources are currently being directed towards increasing the competency of MHPs and support staff working in the community to provide community-based interventions and case management for the general population.

Until recently, Singapore mainstream mental health services and programmes in the community excluded people with intellectual disabilities. Kwok and Chui (2008) found that most Asian countries offer varying standards of generic mental health care to people with intellectual disabilities, often using treatment approaches such as restraints, psychotropic medication and behavioural interventions. Wei et al. (2012) argues that the hospitals in Singapore have become a ‘dumping ground’ (p. 431) for people with intellectual disabilities because they become too difficult to be taken care of in the community by their families. Studies have shown that the deterioration of mental health problems in people with intellectual disabilities can contribute to a significant increase in care burden which may leave family carers feeling overwhelmed and distressed (Dawson et al., 2016; Kim, 2017).

Additionally, there has been a lack of coordination of services for this population in Singapore, resulting in frequent inpatient admissions during which their mental health problems and challenging behaviours were not treated appropriately (Wei et al., 2012). The poor recovery outcomes increased the care burden of family carers and affected participation in the community where adults with intellectual disabilities were denied mainstream services and employment opportunities (Poon, 2015).

More recently, Singapore has established specialist intellectual disability mental health service to address these service gaps and improve the mental health outcomes of people with intellectual disabilities. This specialist service consists of a multidisciplinary team comprising a psychiatrist, case manager, psychologist and occupational therapist, located in a tertiary psychiatric hospital, who offer inpatient and outpatient services for adults with neurodevelopmental disorders, including intellectual disabilities. These professionals have had additional training and experience in working with this population. Adults with intellectual disabilities make up the largest percentage of cases and the majority of them present with low mood or psychotic symptoms as well as aggression towards others and self-injurious behaviours (Moon et al., 2020).

### Aims of study

The current study aims to gain insight into the roles of the MHPs in the specialist intellectual disability mental health service, how they work with other stakeholders to meet the mental health needs of adults with intellectual disabilities and how the current service may be improved.

## Method

### Participants

Participants are referred to as Specialist Mental Health Professionals (SMHP) and the corresponding number allocated to them in Table 1. Inclusion criteria were: (a) MHPs working the hospital for a minimum of the past 6 months in the specialist mental health service and (b) have direct contact with people with intellectual disabilities in their work.

**Table 1.** Demographics of participants.

Participant (SMHP)	Age range	Gender	No. of years of work experience	Current years of work experience with peoplewith intellectual disabilities
1	30–40	F	5–10	>1
2	30–40	M	>10	>5
3	20–30	F	<5	<5
4	30–40	M	5–10	>1
5	20–30	F	<5	<5
6	30–40	M	>10	>5
7	30–40	M	<5	>1
8	20–30	F	<5	>1

Eight (four men and four women) staff from specialist intellectual disability mental health services aged 25 to 38 years (median age 32) were recruited from the hospital. Only SMHP 6 had a professional postgraduate qualification in intellectual disabilities, the others had obtained their skills through on-the-job learning and exposure. SMHP 2 and 5 attended basic training in knowledge of intellectual disabilities and adaptation of therapeutic interventions for this group. Majority of the participants consisted of non-medical staff (i.e. nurses, occupational therapist, social worker and psychologists) and one participant was a psychiatrist. The professions of the participants were not included in the table due to privacy reasons.

### Ethics

The study received ethical approval from University of Birmingham in the UK (Reference No.: ERN_17-1730) and the National Healthcare Group Domain Specific Review Board (Reference No.: 2018/00956) in Singapore. Staff were provided with a study information sheet, detailing the study aims and their rights as a participant. The interviews were carried out by the first author which lasted up to 50 minutes and all participants gave consent for the interviews to be audio recorded.

### Data collection

Potential participants that met the inclusion criteria were provided with an invitation letter that contained brief information about the aims and design of the study. They were informed that their participation in the study was voluntary and would not have an impact on their work performance. Potential participants contacted the first author to arrange an individual face-to-face interview.

Participants were provided with the consent form before the start of the interview and were asked to provide the demographic information. The first author carried out the interviews in English which lasted up to 50 minutes after written consent had been obtained. Participants were asked to talk about their experiences of interacting with people with intellectual disabilities in their work and their own emotional reactions. Additionally, they were asked to provide recommendations to improve the specialist mental health services. The questions (Table 2) that made up the semi-structured interview were informed by previous literature in this area of research. They address the issues and challenges that the SMHP participants face while working with people with intellectual disabilities, their views about the specialist mental health services and public reaction towards this population in the community.

**Table 2.** Interview schedule.

Example of questions
1. What is it like working with people with intellectual disabilities?
2. What makes someone suitable to work with this people with intellectual disabilities?
3. When you are working with people with intellectual disabilities, who do you mostly work with?
4. Do you think there is a difference in how people with intellectual disabilities are being cared for by professional staffs and family members?
5. What do you think about involving individuals with intellectual disabilities having a say about how they wish to manage their health?
6. What do you think about giving individuals with intellectual disabilities a say in their own care management?
7. What do the people and family members find helpful when engaging with your services?
8. What could help you do to improve your clinical practice?
9. How can healthcare professionals improve the quality of life of individuals with intellectual disabilities?

### Analysis

The interviews were transcribed verbatim and analysed using thematic analysis (Braun and Clarke, 2006). The first author carried out the analysis and themes were identified by thorough reading and rereading of the interview transcripts to familiarise himself with the data. In the initial stage of the analysis, the first author derived the key themes and concepts in each interview separately. Subsequently, key themes were collated, categorised and labelled to best describe each of the participants’ narratives. The second stage involved analysing the key themes across the participants’ narratives through identifying similar patterns and connections. Thus, central themes and subthemes emerged (as shown in Table 3). The interpretations of the data were discussed between the authors and were grounded in the original data. An audit of two transcripts was carried out independently by the other authors. The final list of the main themes was discussed among the authors and the quotes from the participants were used to ground the findings in the data. Good agreement was found among the other authors.

**Table 3.** Main themes and sub-themes derived from the analysis.

Main themes	Sub-themes	Participants contributing to the themes
1. Identifying roles	i. My work is challenging	1, 2, 3, 4, 7, 8
	ii. Building a therapeutic relationship	1, 2, 3, 4, 5, 6, 8
	iii. Desirable staff attributes	1, 3, 4, 5, 7, 8
2. Ensuring continuity of care	i. Involving family carers during the treatment process	1, 2, 3, 4, 5, 6, 7, 8
	ii. Working with residential support staff	1, 2, 3, 4, 6, 7, 8
3. Disempowerment of service users	i. ‘*They should be given more choices*’	1, 2, 3, 4, 5, 6, 7
	ii. ‘*The public just want to get away from them*’	2, 3, 4, 5, 6, 7, 8
4. Improving clinical practice		1, 2, 3, 4, 5, 6, 7, 8

The first author is a qualified clinical psychologist with work experience in intellectual disability mental health services in Singapore. This helped him to understand the issues and challenges the participants faced working with this population. A journal was used to bracket his assumptions, thoughts and perspectives which were discussed with the other authors.

## Results

Analysis of the eight interviews resulted in the identification of four main themes and a number of subthemes (see Table 3).

### Main theme 1: Identifying roles

This theme describes the roles and responsibilities of SMHPs working with adults with intellectual disabilities and mental health problems. SMHPs shared how they felt and the challenges faced working with this group.

#### Sub-theme 1: My work is challenging

SMHPs identifed feeling uncertain and fearful when they first started to work in the specialist service, ‘The first experience can be quite daunting, can be quite erm.scary at times’ (SMHP 1). Some admitted feeling uncertain about communicating with adults with intellectual disabilities and needing more effort to observe the person,

I find it very difficult to understand their thought process, or maybe sometimes what they are trying to communicate, I don’t really know…. (SMHP 3)

Some SMHPs identified themselves as a caretaker or authoritative figure to their clients by providing basic step-by-step instructions, ‘I feel like I’m a mother to them. I need to tell them what to do and what to take care of’ (SMHP 8), while others described their role as supporting and including the carers during the treatment sessions to equip them with the necessary skills,

I would be working with their caregivers more…empowering and journeying with them in this journey of helping them to find suitable services, addressing caregiving or parenting issues. (SMHP 7)

Regardless, SMHPs shared their work within a team, consulting with each other and making collaborative treatment decisions together in order to provide a high level of care,

We all work as a team to help the patients. To help the patients to provide the best care that we can have to them. We support each other and collaborate so that we can provide the best to the patient. (SMHP 2)

SMHPs described enjoying their work, ‘…the job it is very fulfilling, like very satisfactory for soul’ (SMHP 5), others reported satisfaction in their work over time especially when their clients improved after treatment,

That fear and daunting feeling will start to decrease as the time goes by. (SMHP 1)

#### Sub-theme 2: Building a therapeutic relationship

SMHPs talked about using different strategies to focus on building a therapeutic relationship or rapport in this second sub-theme, for example engaging the interests of their clients,

I am engaging them with activities, like games, puzzles, colouring, drawing., I would ask their family and caregivers, what does this individual like? What’s their interest in? (SMHP 3)

SMHPs paced their intervention according to the client’s level of engagement. They relied on non-verbal cues to identify any signs of distress, using ‘lots of gestures and visuals without using words and full sentences’ (SMHP 1) to connect with them and check whether they wished to continue with the session.

SMHPs relied on basic visual aids with short verbal instructions, such as developing a communication passport to help other staff understand their clients with intellectual disabilities better,

Usually at the start, I will do a mixture of some of the behavioural interventions, we will use visual aids, we will use short and simple instructions to go through what they did and we will get them to follow and do tasks together…. (SMHP 8)

Additionally, SMHPs felt that it is important to spend more time with the person and use non-verbal communication approaches to build rapport with them. However, there were concerns raised that spending too much time with a particular client may affect their workload and colleagues may perceive that they are not working productively and will be viewed as ‘*lazy*’,

If I spend one whole day to do activity with one profound ID patient [*sic*], I think nobody is going to praise me that much because I never do the rest of my work. I think that might be a very big challenge. (SMHP 2)

#### Sub-theme 3: Desirable staff attributes

In this third sub-theme, many of the SMHPs stressed that having a passion for or interest in working with this specific group is an important prerequisite,

You will definitely have to have a heart (laughs). Passion. (SMHP 7)

The importance of having passion was seen as more important than the need to have prior skills and knowledge as it helps to alleviate some of the negative feelings or challenges that they encounter in their work,

Those who are willing to come and work with…they have compassion, they have this passion to work with disabilities…I think the most important part is to be interested to work with them, then along the way the skills can also learn later. (SMHP 8)

SMHPs also spoke of the importance of being prepared and flexible to deal with unpredictable situations and with challenging behaviours that the adult person with intellectual disabilities might display. They also felt that safety issues were something that mental health professionals should consider before joining this field. Hence, being calm and composed when dealing with an unpredictable and potentially aggressive situation was a necessary quality that SMHPs agreed on,

They should consider about the safety issue, whether they are comfortable to deal with population who are more likely to be unpredictable compared to the general population…. (SMHP 5)

SMHPs also reflected on work culture and stated that it is important to have the skills to ‘work with different people, like allied health and also the patient’s family members to work together. Not only focus on the ID patient [*sic*], but also work with other people together’ (SMHP 4). They viewed having patience as essential when working with this population, taking time to talk to the person, being tolerant of their challenging behaviours and to moderate their expectations of positive outcomes in the short-term.

### Main theme 2: Ensuring continuity of care

This theme reflects the difficulties SMHPs faced when engaging with different stakeholders and their experiences of helping the person to settle back into their communities.

#### Sub-theme 1: Involving family carers during the treatment process

The level of involvement of familys was vital to the delivery of care because SMHPs acknowledged that ‘…the family unit is very important in the Asian culture, most of them live with their family and unable to live independently’ (SMHP 4). The family unit was viewed as providing ‘one-to-one care’ and carers ‘…know a little bit more about the patients themselves and actually gives us a lot more insight of their family member’s behaviours at home’ (SMHP 1), hence SMHPs often work with them to ensure compliance of strategies after discharge.

Some SMHPs seemed cautious and raised concerns that carers may become overly protective, such as assisting their relative to perform daily tasks or avoid bringing them out in the community,

Parents are usually more “helpful” because they know their child has some disabilities, so they want to do everything for them and it is easier to do that rather than training them to be independent as it saves a lot of time. Sometimes the parents do a lot for them so in the end, they lose a lot of skills. (SMHP 8)

Sometimes SMHPs faced resistance from carers who were not willing to engage with them, ‘…they are not very open to suggestions from healthcare professionals, so sometimes, they feel like they know their own child best…that’s when they are very guarded and get aggressive with us’ (SMHP 3). In some instances, SMHPs seemed bewildered as to why some carers abandoned their relative with intellectual disabilities in the hospital and refused to remain in contact with them. They surmised that the demands of taking care of the person and managing their behaviours had become too challenging that the carers decided to cut all ties with the person,

They kind of abandon their beloved ones, their child here with us for so long…there was a promise from you that after a few months, you will bring them back but after that it didn’t happen and they became uncontactable or even disappeared…. (SMHP 2)

Regardless of the negative experiences they encountered with the carers, SMHPs remained adamant that the important goal was for the person to be discharged back to them because of the relationships and bonds in the family which they considered to be protective factors for the person with intellectual disabilities,

I think for family members right, they are very close to the patient, they really understand the patient’s behaviour and they understand how to manage. Maybe if you give them some caregiver training and share the strategies to them, they are able to take care of the patient. (SMHP 6)

Despite the challenges, SMHPs noted positive outcomes and experiences when the carers did find the services useful,

it is difficult to involve the parents, but those who are involved…I can see better prognosis. (SMHP 5)

#### Sub-theme 2: Working with residential support staff

SMHPs highlighted challenges when working with residential support staff, dealing with their scepticism and refusal to admit them into residential homes,

They may want to tell us the things we are trying to implement is not going to work in my environment…so those cases that come back to us or ask us to see again after they are discharged…. (SMHP 1)

Others stated that long-stays in the hospital were not ideal due to physical limitations of the environment and restricted schedules that people with intellectual disabilities had to adhere to, preferring them to stay at the residential insituation,

It’s like everybody is “locked up” in one small area in the hospital for 45 people staying together and they don’t have much freedom. (SMHP 2)

There were mixed views regarding the competency of support staff. Some considered the support staff in residential institutions ‘*well-trained*’ with appropriate strategies to deal with behavioural issues,

They can help patient with behavioural issues, how to reinforce the positive behaviour, how to minimise or reduce the negative behaviour. (SMHP 4)

However, others felt that there was an inconsistency in the level of training that impacted on their attitudes and perception of people with intellectual disabilities,

The staff in disability home or nursing home still lacks training. They don’t have much experience in taking care of this group of people. (SMHP 6)

SMHPs noted that a shortage of manpower in residential institutions would affect the training opportunities for support staff,

Some of the residential staff at the homes struggle to be trained due to manpower issue. Because if you want to train them, it takes a lot of hard work. (SMHP 8)

Despite that, they stressed the need to engage with residential support staff to provide on-going training to improve their attitudes and competency.

### Main theme 3: Disempowerment of service users

This theme outlines the barriers towards empowering the people with intellectual disabilities and SMHPs’ views on integrating them in the community.

#### Sub-theme 1: ‘They should be given more choices’

Most of the SMHPs concurred that adults with intellectual disabilities should be provided with opportunities to be more independence and have autonomy,

Because he can think, he can verbalize to you what he wants, what he prefers so if that’s the case, we should respect the patient’s feelings, we should give enough autonomy. (SMHP 2)

However, adults with moderate to severe intellectual disabilities were often presumed to lack capacity to make decisions related to their care, hence their views were ignored when SMHPs discussed the care plan with family members,

They lacked mental capacity to decide their own care so we usually discuss with the family members and the care and implementation of the treatment is mainly lies with the family members. (SMHP 4)

SMHPs would try to find a common ground between the choices of the person and their family members,

Caregivers would like to have certain plans. Patients may not want it. So what’s important is having a conversation on this with both of them, see what will be in their best interest and to allow our patients and our caregivers to leave with an enhanced quality of life. (SMHP 6)

Some found it emotionally difficult to deny their requests or choices,

Breaking up that news is a bit difficult for me if you asked me in terms of their wants. (SMHP 5)

Despite their strong views on respecting the autonomy and capability of the person with intellectual disabilities, SMHPs felt that in reality, adults with intellectual disabilities had very little choice in where they wished to go after being discharged from the hospital or in having interpersonal relationships with the opposite gender. They noted that family carers restricted the opportunities of their relative due to perceived fear of being stigmatised by the public and wanting to protect them,

They [people with intellectual disabilities] in their adulthood are very motivated to have romantic relationships, to have partners…but I think the difficulty is that sometimes to get them back to reality that they may or they may not end up in a romantic relationship. (SMHP 5)

SMHPs reported concerns that mainstream staff without any knowledge of intellectual disabilities or prior work exposure might have negative attitudes towards people with intellectual disabilities, believing that they were unable to recover from their mental health problems,

Sometimes, they will think that their aggressive concern is part of the personality. They may feel that patients with ID [*sic*] always will have challenging behaviours. They will also be thinking that this group of people is a burden of the society and hopeless. (SMHP 6)

#### Sub-theme 2: ‘The public just want to get away from them’

SMHPs reflected that in Singapore, as in many other countries, adults with intellectual disabilities and mental health problems were not provided with adequate support to allow them to integrate in the community,

Our local culture is not so accommodating. They are not well respected. People have a bias against them. (SMHP 2)

This stigmatisation sometimes created challenges to find appropriate community resources,

Some of our patients have behaviours so they are restricted from community services. They may cause caregivers to feel rejected. They are tired of navigating the services. (SMHP 7)

SMHPs described how this group remained ostracised and segegrated from the community,

The public just want to get away from them and look at them very briefly. They don’t support people with ID [*sic*] to stay in the community, rather prefer them to stay in the disability home or hospital or stay at home, not to mix with the public. (SMHP 6)

People with intellectual disabilities, as noted by SMHPs, were sometimes treated harshly by the public when the public perceived their behaviours as a nuisance and dangerous,

I think in terms of public attitudes, some can be very not welcoming…like when they make noise in the bus…they will be told off to alight at the next bus stop. Whenever they have aggressive behaviour, usually the police will be involved, handcuff them and bring them which sometimes cause trauma for the patient. (SMHP 5)

The majority of the SMHPs acknowledged more work was required to change public perception towards this population, however they noted that current knowledge and understanding of disability in Singapore was new and developing, thus were hopeful that there would be a positive change in societal attitudes and perception through reseach and public campaigns,

I think the stigma exists and from my experience, general public may even have more negative view for patient with ID [*sic*]. we need to address the stigma, that’s why we have a lot of campaigns to address the stigma. (SMHP 4)

### Main theme 4: Improving clinical practice

SMHPs discussed ways to improve their clinical practice and service delivery in this theme. They shared that they would like to have more time to carry out their work with this service group given the complexity of their mental health problems,

More time is needed…this kind of work cannot be rushed…. (SMHP 5)

Ongoing supervision and consultation with other colleagues in the team were beneficial for the SMHPs however there was a lack of experienced staff because of high turnover,

It’s usually peer support rather than mentoring or supervision because the turnover rate of the staff is high, you don’t really have a lot of experienced staff here to support you with your clinical development. (SMHP 1)

Having a support group was perceived as useful to allow them to express their emotions in a safe place without the fear of being judged. Or a more experienced colleague as mentor providing emotional support to newly hired staff,

I think support group is really needed for the clinicians also…this support group, just to not to talk about any goals but to vent out for emotional support. (SMHP 5)

They discussed about how the services could be adapted to focus on empowering people with intellectual disabilities to make choices. They felt that there should be paradigm shift in how they provide services, for example moving towards ‘supporting them to make a decision by themselves rather than just making a decision for them. We must find out what is the best interest’ (SMHP 6). They shared that the service delivery model should consider the social relationships of people with intellectual disabilities, which was currently difficult for them to address due to a lack of awareness, ‘I think that is my difficulty in terms of working…the social relationships…it’s very hard to convince or tell people actually what are you dealing with or what is this person like?’ (SMHP 5). One of the SMHPs raised concerns whether it was acceptable within their culture to facilitate autonomy and decision-making in adults with intellectual disabilities,

We even invited an ID [*sic*] expert from UK, he was even sharing that two mild patients, a male and one female, they ask him can we make love tonight? Then he answered…you are big enough to make your own decision. I was very shocked to hear that. I don’t think this is possible to have one ward with males and females are together. (SMHP 2)

## Discussion

Individual interviews with this specific sample of SMHPs in Singapore had provided a deeper understanding of the specialist intellectual disability mental health services and SMHPs’ experiences, views and feelings working with this specific population. This study is unique in exploring the role and work responsibilities of SMHPs in an Asian context. Most current literature is focused on MHPs working in western countries with individualistic values that promote autonomy and empowerment in people with intellectual disabilities. MHPs in Singapore encounter challenges when adopting westernised philosophies of individualism and normalisation in their practice as the family often has the final say on how their relatives should live their lives. The current findings indicate high levels of stigma may affect MHPs’ efforts to integrate people with intellectual disabilitiesinto their communities and when family members are unwilling to continue caring for them, the preferred option is often admission to a residential home, as a way of segregating them.

In the theme ‘*Disempowerment of service users*’, there is a wrangle between SMHPs’ views of promoting autonomy and empowerment in people with intellectual disabilities and the practical limitations in achieving this in a collectivist culture where the family unit plays a crucial role in making decisions for the person. Kanter (2012) argued that people with disabilities in the US have the legal right to decide where and with whom they wish to live in the community under the Convention on the Rights of People with Disabilities adopted by the United Nations. Findings in the literature suggest that a strong cultural preference to house them in residential homes segregated from the community (Scior et al., 2010) could explain the limited choices for people with intellectual disabilities to have a say over their treatment plan and their lives. MHPs in Singapore are similarly likely to find it difficult to challenge the cultural norms as described in the sub-theme ‘*Involving family carers during the treatment process*’ and instead choose to work within the cultural boundaries until such time when society is more accepting of this group living in community-based supported accommodation housing.

Almost all the SMHPs interviewed for this study recognise the importance of building rapport, having a therapeutic relationship and patience when working with people with intellectual disabilities as described in the first theme ‘*Identifying roles*’. Yet most previous research has focused on the competency and training needs of MHPs to increase their ability to care and manage the behavioural problems of people with intellectual disabilities without considering the importance of building therapeutic relationships on clinical outcome (Sajith et al., 2017; Werner et al., 2013; Wilkinson et al., 2012). Participants in the study discussed the need for ongoing supervision and training as reflected in the theme ‘*Improving clinical practice*’. Hence, having the competency to provide and adapt talking therapies to meet the needs of people with intellectual disabilities should be promoted as best clinical practices to improve the current specialist intellectual disability service. Focusing on these skills could facilitate the therapeutic process of building trust and rapport with service users which may translate to increasing their motivation and readiness to change during the treatment process (Bonell et al., 2012; Mason, 2007; Willner, 2005).

Living in residential institutions becomes the only alternative for people with intellectual disabilities when their family are unavailable or unwilling to take them back after discharge from the hospital. Thus, one of the learning points from this study is that SMHPs emphasise the need to involve the family carers to ensure positive outcomes for people with intellectual disabilities as described in the theme ‘Ensuring continuity of care’. Family carers experience a loss of control over their lives when they are unable to manage their relatives’ challenging behaviours, which may contribute to high psychological distress (Dawson et al., 2016; Waite et al., 2017). Carers in Singapore may lack the coping strategies to address their relative’s challenging behaviours and emotional difficulties, and following their relative’s admission to the specialist mental health intellectual disability service for treatment they may fear their relative displaying the same problems after discharge. SMHPs in the study did not mention that they offered psychological interventions for family carers. Thus, it is recommended that SMHPs are trained and supported to understand the emotional struggles that family carers often experience and provide resources for families to be able to receive and benefit from appropriate psychological help and comprehensive care plans (Howatson, 2005) before as well as after discharge from the hospital.

The sub-theme ‘*Working with residential support staff*’ describes an important component of SMHPs’ work which is collaborating with residential support staff and providing them with training and supervision to increase their skill competency and willingness to care for people with intellectual disabilities and mental health problems. Research findings in an East Asian context have shown the effectiveness of staff training to change attitudes towards facilitating the self-determination of people with intellectual disabilities (Wong and Wong, 2008). SMHPs in the current study noted the mixed reactions from support staff and a lack of collaborative practice and understanding between both parties. The organisational culture (Hatton et al., 1999) of the residential institution may affect the level of engagement between support staff and SMHPs (Rose et al., 2006). Support staff’s attributions of the challenging behaviours in people with intellectual disabilities have been found to have an impact on the quality and delivery of care (Williams and Rose, 2007), which may also account for staff’s (un)willingness to work with SMHPs. Applied psychologists have identified the importance of building therapeutic relationships with staff in a residential setting and noted that some staff were hesitant to share information with them (Stenfert Kroese and Smith, 2018), also observed by the SMHPs in the current study, SMHPs need to consider interpersonal and organisational factors when working with support staff in the community to order to implement behavioural programmes successfully.

In the theme ‘*Improving clinical practice*’, SMHPs identified a common challenge to provide services that promote autonomy and empowerment in adults with intellectual disabilities and contrasted the differences in attitudes among MHPs in western and non-western societies. Findings from cross-cultural research reveal that non-western countries, when compared to western countries, have less favourable attitudes towards empowerment of people with intellectual disabilities and are less inclined to provide the same rights to them as to the general population (Benomir et al., 2016; Patka et al., 2013; Scior et al., 2010). These findings further suggest that non-western people having negative attitudes and views regarding sexuality issues and sexual rights of people with intellectual disabilities when compared to westerners (Sankhla and Theodore, 2015) and across the world people with intellectual disabilities have fewer social networks and lead a more impoverished lifestyle than the general population (Lippold and Burns, 2009). The role of culture appears to have a significant impact on the public’s and MHPs’ attitudes towards the sexuality of people with intellectual disabilities (Ditchman et al., 2017; Meaney-Tavares and Gavidia-Payne, 2012). Finding ways to address the quality-of-life domains of self-determination and interpersonal relations in people with intellectual disabilities continues to be a challenge in most cultures (Morisse et al., 2013).

### Recommendations

The findings of this study indicate that besides providing more training, opportunities for overseas work experience and supervision by experienced clinical staff, there is a need to move beyond improving the competencies of healthcare professionals and also consider policies to improve the hiring and retention of staff. Government agencies may also consider the social and housing needs of people with intellectual disabilities instead of narrowly focusing on their health and employability. This may involve public campaigns to create awareness in the public of their human rights. Improving freedom to choose could be achieved by incorporating views of people with intellectual disabilities in care plans. Community presence and participation (O’Brien, 1992) could be encouraged by developing support plans for people with intellectual disabilities that facilitate integration and community participation and widen their social network.

Outcome measures that are comprised of interpersonal and empowerment domains for people with intellectual disabilities could be developed to evaluate the progress made in these areas (Flynn et al., 2017). Support staff in the community or residential settings and mainstream primary healthcare professionals can take a more active role in screening and assessment of mental health problems with support and supervision from experienced SMHPs. Community-based therapeutic interventions should be made available in residential settings and support staff should be equipped with the necessary skills and competency to manage and address the mental health issues for the residents with intellectual disabilities (Dodd et al., 2013).

Lack of organisational support is a common reason for staff’s lack of work satisfaction and affects the quality of intellectual disability services (Hermsen et al., 2014; Innstrand et al., 2004). It is, therefore, imperative that hospital management and policy makers listen to MHPs’ concerns about their high work demands and provide adequate resources to support them through, for example, making changes in the recruitment process and providing interventions to address stress and burnout (Edwards and Burnard, 2003).

SMHPs in the current sample work in a multi-disciplinary team with carers and support staff in the community as stakeholders instead of working one-to-one with people with intellectual disabilities. It is therefore important that SMHPs build therapeutic relationships with these various stakeholders to ensure that the treatment plan is agreed by all and they are willing to implement the intervention strategies consistently across different settings. Hence, having the necessary interpersonal characteristics, such as honesty and warmth are important for building genuine and trusting relationships. Thus, these desirable qualities must be assessed when considering suitable candidates to work in specialist intellectual disability mental health services.
